# Extracellular CIRP-Impaired Rab26 Restrains EPOR-Mediated Macrophage Polarization in Acute Lung Injury

**DOI:** 10.3389/fimmu.2021.768435

**Published:** 2021-12-01

**Authors:** Wen Zhang, Yao Wang, Chuanwei Li, Yu Xu, Xia Wang, Di Wu, Zhan Gao, Hang Qian, Zaichun You, Zhiren Zhang, Binfeng He, Guansong Wang

**Affiliations:** ^1^ Institute of Respiratory Diseases, Department of Pulmonary and Critical Care Medicine, Xinqiao Hospital, Third Military Medical University, Chongqing, China; ^2^ Department of Cardiology, Daping Hospital, Third Military Medical University, Chongqing, China; ^3^ Department of General Practice, Xinqiao Hospital, Third Military Medical University, Chongqing, China; ^4^ Institute of Immunology, Third Military Medical University, Chongqing, China; ^5^ Department of Pulmonary and Critical Care Medicine, Zhongshan Hospital, Fudan University, Shanghai, China

**Keywords:** acute lung injury (ALI), eCIRP, EPOR, Rab26 GTPase, macrophages

## Abstract

Acute lung injury (ALI)/acute respiratory distress syndrome (ARDS) is a condition with an imbalanced inflammatory response and delayed resolution of inflammation. Macrophage polarization plays an important role in inflammation and resolution. However, the mechanism of macrophage polarization in ALI/ARDS is not fully understood. We found that mice with lipopolysaccharide administration developed lung injury with the accumulation of extracellular cold-inducible RNA-binding protein (eCIRP) in the lungs. eCIRP, as a damage-associated molecular pattern (DAMP), inhibited M2 macrophage polarization, thereby tipping the balance toward inflammation rather than resolution. Anti-CIRP antibodies reversed such phenotypes. The levels of macrophage erythropoietin (EPO) receptor (EPOR) were reduced after eCIRP treatment. Myeloid-specific EPOR-deficient mice displayed restrained M2 macrophage polarization and impaired inflammation resolution. Mechanistically, eCIRP impaired Rab26, a member of Ras superfamilies of small G proteins, and reduced the transportation of surface EPOR, which resulted in macrophage polarization toward the M1 phenotype. Moreover, EPO treatment hardly promotes M2 polarization in Rab26 knockout (KO) macrophages through EPOR. Collectively, macrophage EPOR signaling is impaired by eCIRP through Rab26 during ALI/ARDS, leading to the restrained M2 macrophage polarization and delayed inflammation resolution. These findings identify a mechanism of persistent inflammation and a potential therapy during ALI/ARDS.

## Introduction

Acute lung injury (ALI)/acute respiratory distress syndrome (ARDS) is a complex clinical syndrome with excessive acute inflammation induced by various stimuli, which are associated with the damaged alveolus–capillary barrier and increased endothelial permeability, resulting in life-threatening hypoxemia (1–3). Macrophages, as the important immune cells, play a key role in regulating inflammatory responses and resolution (4–6). After being stimulated by damage-associated molecular patterns (DAMPs) or pathogen-associated molecular patterns (PAMPs), macrophage polarization is shifted to an M1 phenotype, accompanied by the secretion of pro-inflammatory cytokines, which recruit lots of neutrophils and inflammatory macrophages to promote and sustain the inflammatory response (6). During the steady-state or resolution of ALI/ARDS, macrophages express mannose receptor (CD206), β-glucan-specific receptor (Dectin-1), and potent scavenger receptors, which help to define an M2, alternative-activated, anti-inflammatory cell activation state (7, 8). Thus, the balance shifting between M1 and M2 macrophages is crucial for inflammatory responses.

DAMPs are released from the necrotic damaged cells. They act as potent activators for triggering a non-infectious and uncontrolled inflammatory response, resulting in organ injury and death (9). Cold-inducible RNA-binding protein (CIRP) is an evolutionarily conserved RNA partner (10). Intracellular CIRP is currently thought to stabilize specific mRNAs and facilitate translation for a survival advantage when cells are under stress. Extracellular CIRP (eCIRP), as a DAMP, is discovered to be present under various inflammatory conditions and could act as a pro-inflammatory factor. Serum CIRP levels were strongly correlated with procalcitonin, interleukin (IL)-6, and C-reactive protein (CRP) levels and linked to the severity of and mortality from community-acquired pneumonia (11). Recombinant CIRP could induce inflammatory responses *in vivo* and *in vitro*, and neutralization of CIRP could attenuate sepsis during fluid resuscitation in hemorrhaged rats (12), indicating that eCIRP might be a potential target for ALI/ARDS therapy. However, the underlying mechanism of eCIRP-induced inflammation remains to be fully understood.

Erythropoietin (EPO) receptor (EPOR) plays an important role in induced hematopoiesis in erythroid progenitor cells when its ligand EPO binds to EPOR and activates downstream signal pathway (13). Further study displayed that EPOR is also located at the cell surface of macrophages (14, 15). EPO could promote the expression of EPOR in macrophages and then suppress inflammatory gene expression (15–19) and promote apoptotic cell clearance (15). Yang et al. (20) revealed that activation of EPOR/Janus kinase (JAK)2/signal transducer and activator of transcription (STAT)3 signaling by EPO suppressed the M1 phenotype and shifted macrophage polarization toward the M2 phenotype in the presence of IL-4. These documents hinted that activation of EPOR-mediated signaling required EPOR location at the cell surface. However, how to transport EPOR to cell surface from cytoplasm pool is still unknown.

Ras superfamilies of small G proteins (Rab GTPases) are well known as the regulators of intracellular trafficking, including the movement of newly synthesized receptors from the endoplasmic reticulum (ER) to the cell surface, endocytosis of receptor–ligand complex from the cell surface, and translocation of the complexes to endosomes (21). Recently, we found that Rab26 deficiency aggravates lipopolysaccharide (LPS)-induced lung tissue injury and enhanced the accumulation of neutrophils in the pulmonary vasculature, alveolar cavity, and pulmonary interstitium (22), suggesting that Rab26 might regulate the inflammatory response in macrophages. However, the mechanism is still unclear.

In this study, we investigated the effect of eCIRP on EPOR signaling and the polarization of macrophages in the ALI/ARDS model. We found that eCIRP reduced the level of EPOR at the cell surface, restrained macrophage polarization to the M2 phenotype, and increased the inflammatory response. Furthermore, eCIRP suppressed the expression of Rab26, and Rab26 deficiency restrained M2 macrophage population and increased the inflammatory response. Rab26 regulated the cell surface expression of EPOR, and EPO treatment could restrain M1 macrophage polarization *via* Rab26/EPOR/peroxisome proliferator-activated receptor (PPAR)γ axis. The novel findings show the new mechanism of eCIRP in ALI, which could be a promising therapeutic target.

## Results

### eCIRP Is Temporally Activated in Acute Lung Injury

**Figure 1:**
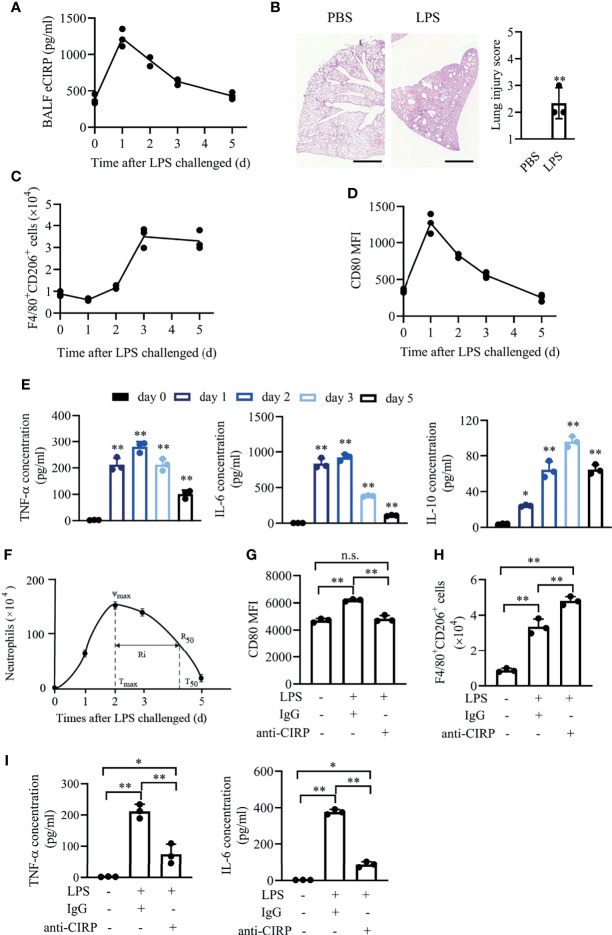
eCIRP is temporally activated in acute lung injury. WT mice (n = 8 for each time point) were treated with 3 mg/kg LPS (i.t.) at the indicated times (days 0, 1, 2, 3, and 5). **(A)** The concentrations of BALF eCIRP were measured by ELISA at the indicated time after LPS administration i.t. **(B)** Lung tissue samples from mice with LPS or PBS administration for 24 h were dissected and subjected to H&E staining. Left panel: representative lung tissue images. Right panel: histopathological mean lung injury scores from each group. Scale bar: 1,000 µm. Measurements were performed in triplicate for data analysis. **P <0.01 *vs.* PBS group. Macrophages (F4/80^+^Ly-6G^-^) in BALF from WT mice were collected at 0, 1, 2, 3, and 5 days after i.t. LPS (3 mg/kg) challenge, and the number of CD206^+^ **(C)** macrophages and the MFIs of CD80 **(D)** in BALF macrophages were measured by FACS. **(E)** The concentrations of secreted TNF-α, IL-6, and IL-10 in BALF at the indicated time were measured. **(F)** The time course of neutrophil (Ly6G^+^F4/80^-^) numbers in BALF and resolution indices was calculated by flow cytometry. WT mice were administered LPS (3 mg/kg, i.t.) and then were treated with IgG or anti-CIRP antibody (1 mg/kg/day) *via* caudal vein for 3 days. Control mice were administered PBS. The macrophages’ MFIs of CD80 **(G)**, the number of CD206^+^ macrophages **(H)**, and cytokine expression **(I)** in BALF at day 3 after LPS treatment were measured (n = 3). Data are representative of at least two independent experiments. Results were expressed as mean ± SD. n.s., not statistically significant., *P < 0.05, **P < 0.01 compared to 0 h group. Statistics: unpaired two-tailed Student’s t-test **(B)**, one-way ANOVA with Tukey’s *post-hoc* test for multiple comparisons **(E, G**–**I)**. eCIRP, extracellular cold-inducible RNA-binding protein; WT, wild type; BALF, bronchoalveolar lavage fluid; ELISA, enzyme linked immunosorbent assay; LPS, lipopolysaccharide; FACS, fluorescence activated cell sorter.

Given that DAMPs are the important concerns in sepsis (9), we first detected the temporal expression of eCIRP in ALI/ARDS induced by intratracheal administration of LPS (3 mg/kg). LPS has been shown to induce eCIRP release and trigger a systemic inflammatory response in sepsis (12). Using the mouse model of LPS-induced ALI/ARDS, we found that instillation of LPS significantly increased eCIRP level in bronchoalveolar lavage fluid (BALF). The level of eCIRP peaked at about day 1 and gradually reduced to background levels at day 5 (**Figure 1A**), indicating that eCIRP is involved in the induction and resolution of ALI. To confirm the pathological changes induced by LPS, lung tissue samples were dissected at day 1 and subjected to H&E staining. A large number of leukocytes accumulated in the lungs after LPS administration (**Figure 1B**). According to the methods shown in **Supplementary Figure S1A**, we found that F4/80^+^CD206^+^ macrophage counts increased steadily from days 2 to 3 and then gradually decreased (**Figure 1C**), while the mean fluorescence intensity (MFIs) of CD80 in BALF macrophages peaked at day 1 and decreased to background levels at around day 5 (**Figure 1D**).

We further observed that the secreted pro-inflammatory cytokine tumor necrosis factor-α (TNF-α) and interleukin-6 (IL-6) in BALF rapidly increased and then decreased at around day 5 (**Figure 1E**). While the anti-inflammatory cytokine IL-10 was also induced, it gradually decreased and remained high at day 5. In this model, BALF neutrophils increased rapidly, peaked at day 2, followed by a gradual decrease to background levels at around day 5, with a resolution interval (Ri, T_50_–T_max_) (19), which is the time when 50% of neutrophils are lost from BALF, of approximately 52.7 h (**Figure 1F**).

To explore the effect of eCIRP on macrophage polarization and inflammation, we performed the *in vivo* eCIRP neutralization assay in LPS-induced ALI/ARDS. Anti-CIRP antibody (1 mg/kg/day) was administered *via* tail vein once a day for 3 days after LPS administration. The MFIs of CD80 in BALF macrophages were increased in wild-type (WT)-LPS mice at day 3, and administration of anti-CIRP antibody significantly reduced CD80 MFIs (**Figure 1G**). The number of CD206^+^ macrophages was further increased in WT-LPS mice given anti-CIRP antibody compared to that in WT-LPS mice given control immunoglobulin G (IgG) (**Figure 1H**). Thus, we found that eCIRP neutralization antibody could attenuate M1 phenotype and enhance M2 phenotype in macrophages. BALF levels of TNF-α and IL-6 were significantly reduced in the group given anti-CIRP antibody (**Figure 1I**). These data suggest that eCIRP might control LPS-induced inflammatory response through regulating macrophage polarization *in vivo*.

### eCIRP Restrains M2 Macrophage Polarization and Induces Inflammatory Response

Next, we sought to investigate the effect of eCIRP on macrophages *in vitro*. WT bone marrow derived macrophage (BMDMs) were treated with various doses of recombinant human CIRP (rhCIRP; 0, 0.1, 1, 10 µg/ml), and these data showed that the mRNA levels of pro-inflammatory factors (TNF-α, IL-6, and IL-1β) and M1 phenotypic molecules (CD80 and CD86) were significantly increased after BMDMs were stimulated with rhCIRP (1 µg/ml) for 24 h. Thus, we set 1 µg/ml rhCIRP for the BMDM stimulation (**Supplementary Figures S1B, C**).

**Figure 2:**
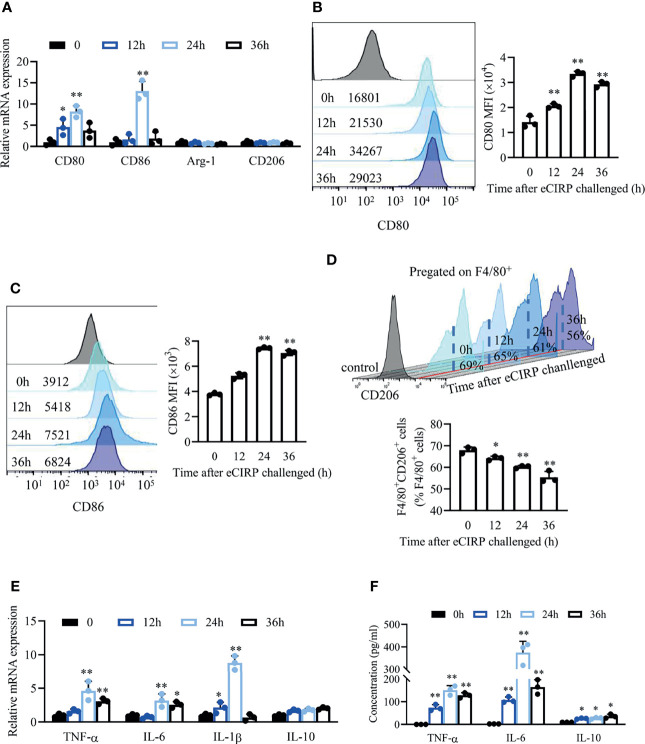
eCIRP impaired/restrained M2 macrophage polarization and induced a pro-inflammatory response. **(A)** A qPCR assay was conducted to evaluate the mRNA expression of CD80, CD86, Arg1, and CD206 in BMDMs after eCIRP treatment for 0, 12, 24, and 36 h (n = 3). The MFI of CD80 **(B)** and CD86 **(C)** and the percentage of CD206^+^ macrophages in BMDMs **(D)** were measured by FACS at indicated time after eCIRP administration (n = 3). **(E)** A qPCR assay was conducted to evaluate the mRNA expression of TNF-α, IL-6, IL-1β, and IL-10 in BMDMs after eCIRP treatment at the indicated time (n = 3). **(F)** The concentrations of TNF-α, IL-6, and IL-10 in the supernatant at indicated time after eCIRP administration were measured. Data are representative of at least two independent experiments. Results were expressed as mean ± SD. *P < 0.05, **P < 0.01 compared to 0 h group. Statistics: One-way ANOVA with Tukey’s *post-hoc* test for multiple comparisons **(A–F)**. eCIRP, extracellular cold-inducible RNA-binding protein; PCR, Polymerase Chain Reaction; MFI, mean fluorescence intensity; BMDM, bone marrow derived macrophage; FACS, fluorescence activated cell sorter.

WT BMDMs were treated with rhCIRP (1 µg/ml) for 12, 24, and 36 h, and the mRNA and cell surface expressions of CD80 and CD86 were significantly increased compared to those of the control group (**Figures 2A** **–C**). The mRNA levels of M2 phenotypic molecules (Arg-1 and CD206) were not influenced after rhCIRP administration (**Figure 2A**). The percentage of CD206^+^ macrophages gradually decreased (from 69% to 56%) with the time of rhCIRP administration (**Figure 2D**). Collectively, eCIRP would promote macrophages to M1 polarization and restrain M2 polarization.

We detected the inflammatory cytokines in BMDMs after rhCIRP administration. The mRNA levels (**Figure 2E**) and supernatant concentration (**Figure 2F**) of pro-inflammatory cytokines TNF-α, IL-6, and IL-1β significantly increased after rhCIRP administration and peaked at 24 h, while the mRNA levels (**Figure 2E**) and supernatant concentration (**Figure 2F**) of anti-inflammatory cytokine IL-10 were induced after rhCIRP administration.

Taking the *in vivo* and *in vitro* data together, LPS-induced eCIRP resulted in increased levels of pro-inflammatory cytokines and restrained M2 macrophage phenotype.

### Myeloid Erythropoietin Receptor Deficiency Aggravates Inflammation in Lung Injury

**Figure 3:**
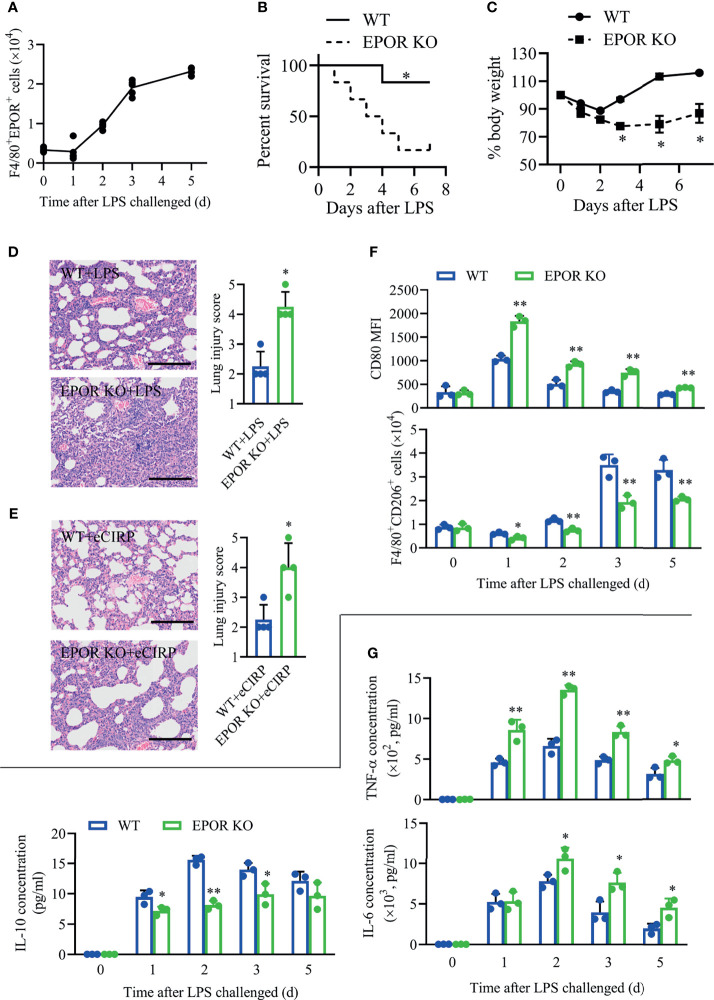
Myeloid EPOR deficiency aggravates inflammation in lung injury. **(A)** WT mice (n = 5) were treated with 3 mg/kg LPS (i.t.) at the indicated times (days 0, 1, 2, 3, and 5). The number of F4/80^+^EPOR^+^ macrophages in BALF was measured by FACS. **(B)** EPOR cKO and WT mice were challenged with 10 mg/kg LPS i.t., and survival was monitored (n = 6). **(C)** EPOR cKO and WT mice were treated with 3 mg/kg LPS (i.t.) at the indicated times. Change in body weight was monitored over 6 days (n = 4). **(D)** Lung tissue samples from mice with LPS administration for 48 h were dissected and subjected to H&E staining. Lung injury scores in each group were analyzed (n = 4). Scale bar: 150 µm. **(E)** EPOR cKO and WT mice were treated with 1 mg/kg eCIRP (i.t.). Lung tissue samples from mice with eCIRP administration for 48 h were dissected and subjected to H&E staining. Lung injury scores in each group were analyzed (n = 4). Scale bar: 150 µm. WT and EPOR cKO mice were treated with 3 mg/kg LPS (i.t.) at the indicated times (days 0, 1, 2, 3, and 5). **(F)** The MFI of CD80 and the percentage of CD206^+^ cells in BALF macrophages were measured by FACS at the indicated time (n = 3). **(G)** The concentrations of TNF-α, IL-6, and IL-10 in BALF at the indicated time was measured (n = 3). Data are representative of at least two independent experiments. Results were expressed as mean ± SD. *P < 0.05, **P < 0.01 compared to the WT group at indicated time. Statistics: Log-rank test **(B)** or unpaired two-tailed Student’s t-test **(C**–**G)**. EPOR, erythropoietin receptor; LPS, lipopolysaccharide; eCIRP, extracellular cold-inducible RNA-binding protein; WT, wild type; BALF, bronchoalveolar lavage fluid; MFI, mean fluorescence intensity; FACS, fluorescence activated cell sorter.

Furthermore, we found that the accumulation of EPOR^+^ macrophages in BALF continuously increased since day 2 (**Figure 3A**) in LPS-induced ALI/ARDS, indicating EPOR signaling might be involved in lung injury. In order to explore the role of EPOR signaling in acute lung injury, the myeloid EPOR_loxp_/loxpLyz2-Cre+/+ mice (EPOR-cKO) mice were generated by crossing LysM-Cre^+/+^ mice with EPOR^loxp^/^loxp^ mice (17). Indeed, decreased animal survival was observed in EPOR-cKO mice compared with WT control mice (**Figure 3B**, 83.3% *vs.* 16.7%, *P* = 0.0196) at day 7 after LPS treatment (10 mg/kg). WT mice exhibited weight gain (**Figure 3C**) from days 2 to 6 compared to EPOR-cKO mice from days 4 to 6. Unlike in WT controls, LPS-induced lung inflammation was aggravated in EPOR-cKO mice according to lung injury scores (**Figure 3D**; 2.25 *vs.* 4.25, *P* = 0.0013). Similarly, EPOR-cKO mice treated with rhCIRP suffered from more severe lung damage than WT mice (**Figure 3E**; 2.25 *vs.* 4.0, *P* = 0.0106).

In the LPS-induced ALI/ARDS model, the MFIs of CD80 on BALF macrophages were higher in EPOR-cKO mice than in WT mice, while F4/80^+^CD206^+^ macrophage counts remained higher in WT mice than in EPOR-cKO mice (**Figure 3F**). Additionally, we observed that the secreted pro-inflammatory cytokines TNF-α and IL-6 in EPOR-cKO mice remained at a higher level than those in WT mice, especially at day 2 (**Figure 3G**), while the level of anti-inflammatory cytokine IL-10 in EPOR-cKO mice was lower than that in WT mice. More severe BALF neutrophil infiltration and more apoptotic neutrophils were observed in EPOR-cKO mice compared to those in control mice (**Supplementary Figures S2A, B**), indicating the important role of macrophage EPOR signaling in the ALI/ARDS model.

### Myeloid Erythropoietin Receptor Is Required for eCIRP-Induced Macrophage Phenotype Shifting

**Figure 4:**
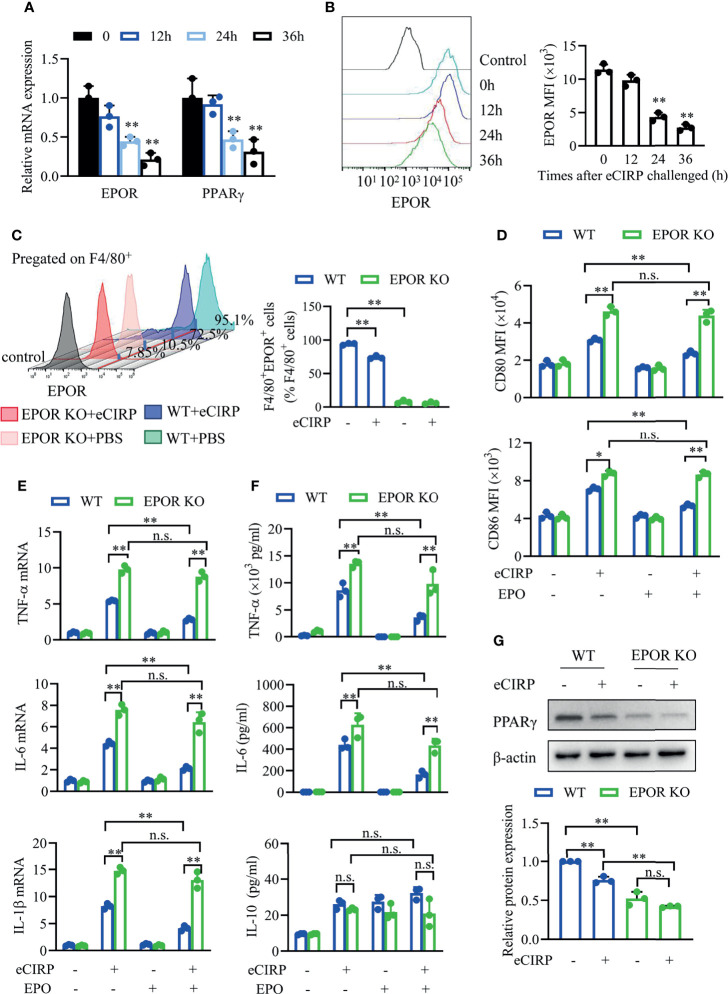
Myeloid EPOR signaling is essential for macrophage polarization. **(A)** A qPCR assay was conducted to evaluate the mRNA expression of EPOR and PPARγ after WT BMDMs were treated with eCIRP for the indicated times (n = 3). **(B)** The MFIs of cell surface EPOR in BMDMs treated with eCIRP for the indicated times were tested by FACS (n = 3). **P < 0.01 *vs.* 0 h group. **(C)** The percentage of EPOR^+^ cells in WT and EPOR cKO BMDMs treated with or without eCIRP for 24 h (n = 3). **(D)** The MFIs of CD80 and CD86 in WT and EPOR cKO BMDMs were measured by FACS with or without eCIRP (1 µg/ml) and rhEPO (20 IU/ml) administration (n = 3). **(E)** A qPCR assay was conducted to evaluate the mRNA expression of TNF-α, IL-6, and IL-1β in WT and EPOR cKO BMDMs with or without eCIRP and rhEPO (20 IU/ml) administration (n = 3). **(F)** The supernatant concentrations of TNF-α, IL-6, and IL-10 in BMDMs were measured with or without eCIRP (1 µg/ml) and rhEPO (20 IU/ml) administration (n = 3). **(G)** Western blot analysis of PPARγ and β-actin protein expression was conducted after WT and EPOR cKO BMDMs were treated with eCIRP for 24 h (n = 3). Data are representative of at least two independent experiments. Results were expressed as mean ± SD. n.s., not statistically significant. *P < 0.05, **P < 0.01. Statistics: One-way ANOVA with Tukey’s *post-hoc* test for multiple comparisons **(A–G)**. EPOR, erythropoietin receptor; PCR, Polymerase Chain Reaction; PPAR, peroxisome proliferator-activated receptor; BMDM, bone marrow derived macrophage; eCIRP, extracellular cold-inducible RNA-binding protein; WT, wild type; EPO, erythropoietin; MFI, mean fluorescence intensity; FACS, fluorescence activated cell sorter.

Previous study showed that EPOR could regulate macrophage polarization (20). We wonder to explore whether EPOR is involved in eCIRP-mediated macrophage polarization shifting. The data showed that the mRNA (**Figure 4A**) and cell surface (**Figure 4B**) expression of EPOR in BMDMs was reduced by ~50% at 24 h after rhCIRP (1 µg/ml) administration, and the mRNA levels of PPARγ (**Figure 4A**) decreased with the time of rhCIRP administration, suggesting that EPOR is involved in eCIRP-mediated macrophage polarization shifting. To further investigate the effect of eCIRP on EPOR, EPOR-cKO BMDMs were identified, as the cell surface EPOR expression was significantly reduced compared with control BMDMs. We found that the percentage of EPOR^+^ macrophages was reduced by ~23.8% in WT BMDMs after rhCIRP treatment for 24 h while it did not significantly change in EPOR-cKO BMDMs (**Figure 4C**).

Subsequently, we tested the cell surface expression of CD80 and CD86 through fluorescence activated cell sorter (FACS) *in vitro*. The MFIs of CD80 and CD86 were higher in EPOR-cKO BMDMs compared to control BMDMs after rhCIRP treatment with or without rhEPO (**Figure 4D**). EPO could reduce CD80 and CD86 expression in WT BMDMs, but it did not work in EPOR-cKO BMDMs after rhCIRP treatment. The mRNA and protein levels of TNF-α, IL-6, and IL-1β were higher in EPOR-cKO BMDMs compared to control BMDMs after rhCIRP treatment, and rhEPO could alleviate the inflammation in the control BMDMs while the pro-inflammatory cytokines were still high in EPOR-cKO BMDMs (**Figures 4E, F** **)**. There is no obvious change in IL-10 level between EPOR-cKO and WT BMDMs during different administrations (**Figure 4F**). Consistent with the mRNA levels of PPARγ, the protein expression of PPARγ was reduced after EPOR knockout, and eCIRP would decrease PPARγ expression (**Figure 4G**). Collectively, eCIRP could induce the restrained M2 polarization through EPOR/PPARγ signaling *in vitro*.

### Rab26 Is Critical for eCIRP-Mediated Inflammatory Response in Acute Lung Injury/Acute Respiratory Distress Syndrome

**Figure 5:**
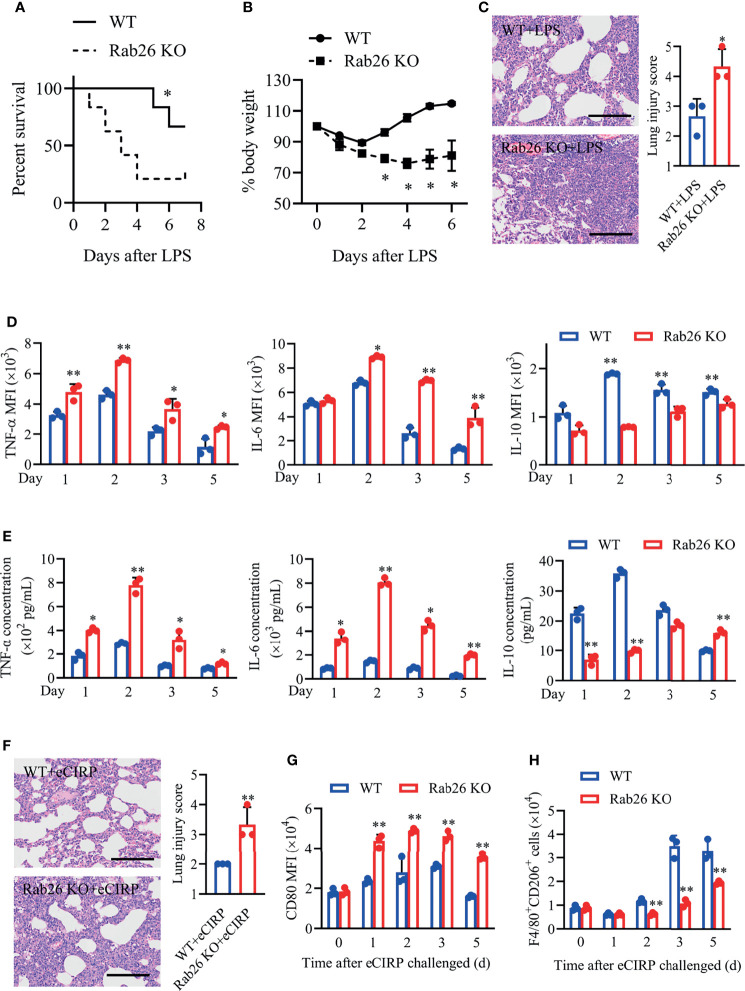
Rab26 is critical for the eCIRP-mediated inflammatory response in lung injury. **(A)** Rab26^-/-^ and WT mice were challenged with 10 mg/kg LPS i.t., and survival was monitored (n = 6). **(B)** Rab26^-/-^ and WT mice were treated with 3 mg/kg LPS (i.t.). Change in body weight was monitored over 6 days (presented as % change from initial body weight) (n = 4 for each group). **(C)** Lung tissue samples from mice with LPS administration for 48 h were dissected and subjected to H&E staining. Lung injury scores were analyzed (n = 3). Scale bar: 150 µm. Macrophages (F4/80^+^) in BALF from WT and Rab26^-/-^ mice were collected at days 1, 2, 3, and 5 after eCIRP (1 mg/kg, i.t.) challenge. The MFIs of intracellular TNF-α, IL-6, and IL-10 in BALF macrophages **(D)** and the concentrations of TNF-α, IL-6, and IL-10 in BALF **(E)** at indicated time were measured (n = 3). **(F)** Rab26^-/-^ and WT mice were treated with 1 mg/kg eCIRP (i.t.) at the indicated times. Lung tissue samples from mice with eCIRP administration for 48 h were dissected and subjected to H&E staining. Lung injury scores in each group were analyzed (n = 3). Scale bar: 150 µm. The MFIs of CD80 in BALF macrophages **(G)** and the number of F4/80^+^CD206^+^ macrophages **(H)** were measured by FACS (n = 3). Data are representative of at least two independent experiments. Results were expressed as mean ± SD. *P < 0.05, **P < 0.01 *vs.* the WT group at indicated time. Statistics: Log-rank test **(A)** or unpaired two-tailed Student’s t-test **(B–G)**. LPS, lipopolysaccharide; BALF, bronchoalveolar lavage fluid; eCIRP, extracellular cold-inducible RNA-binding protein; WT, wild type; MFI, mean fluorescence intensity; FACS, fluorescence activated cell sorter.

Previous reports have revealed that several Rab GTPases, such as Rab37 and Rab8a, play important roles in skewing macrophage polarization and inflammatory response (23, 24). Thus, Rab26^−/−^ (Rab26 KO) mice were generated to explore the role of Rab26 on inflammation during lung injury *in vivo*. Indeed, decreased animal survival was observed in Rab26 KO mice compared with WT control mice (**Figure 5A**; 66.7% *vs.* 16.7%, *P* = 0.028) at day 7 after LPS treatment. WT mice exhibited weight gain (**Figure 5B**; 88.2% to 113%, *P* < 0.05) from days 2 to 6 compared to Rab26 KO mice (74.3% to 82.4%) from days 4 to 6. Unlike in WT controls, LPS-induced lung inflammation was aggravated in Rab26 KO mice according to lung injury scores (**Figures 5C**; 2.67 *vs.* 4.33, *P* = 0.024).

Similarly, Rab26 KO mice with rhCIRP treatment aggravated lung damage (**Figure 5F**; 2.0 *vs.* 3.33, *P* = 0.016), increased the expression of pro-inflammatory cytokines TNF-α and IL-6, and suppressed the expression of IL-10 in intracellular and extracellular levels (**Figures 5D, E** **)**. Besides, the MFIs of BALF macrophages CD80 were significantly higher in Rab26 KO mice than in WT mice (**Figure 5G**), while the number of CD206^+^ macrophages from BALF was lower in Rab26 KO mice than that in WT mice (**Figure 5H**) during eCIRP-induced ALI/ARDS. These findings demonstrated that Rab26 deficiency restrained M2 macrophage polarization and aggravated the inflammatory response, indicating that Rab26 is a critical mediator for eCIRP-induced inflammatory response during ALI *in vivo*.

### Rab26 Is Critical for Macrophage Polarization

**Figure 6:**
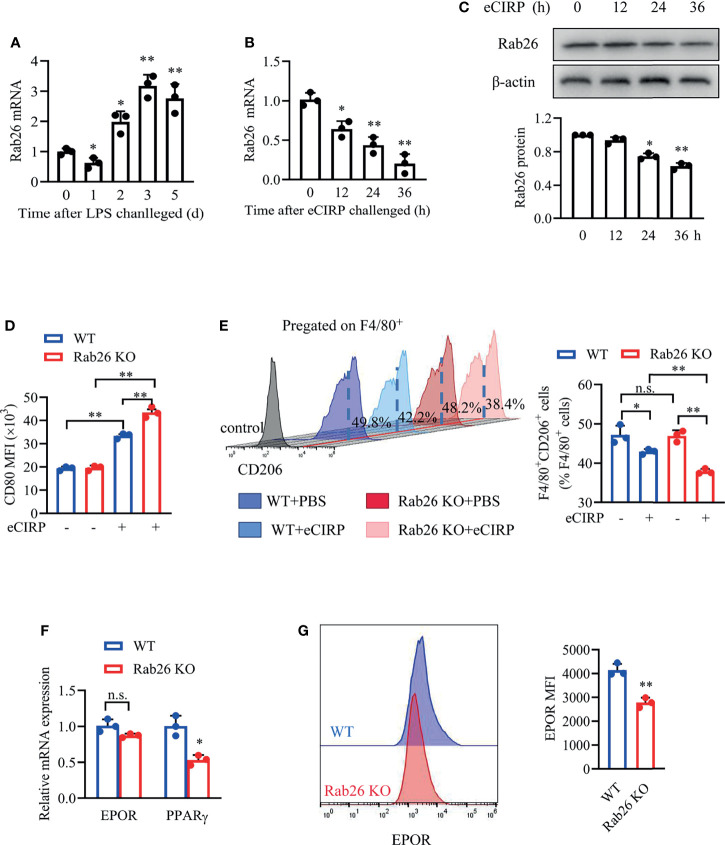
Rab26 is critical for macrophage polarization. **(A)** Macrophages in BALF from WT-ALI mice (3 mg/kg LPS i.t.) were isolated by magnetic-activated cell sorting with F4/80 labeled, and qPCR analysis of the Rab26 mRNA expression in BALF macrophages was tested at the indicated times (days 0, 1, 2, 3, and 5) (n = 3). *P <0.05, **P <0.01 *vs.* the day-0 group. A qPCR assay was conducted to evaluate the mRNA expression of Rab26, **(B)** and Western blot analysis of Rab26 and β-actin protein expression **(C)** was conducted after WT BMDMs were treated with eCIRP for the indicated times (n = 3). *P < 0.05, **P < 0.01 *vs.* 0 h group. The membrane CD80 MFI of BMDMs **(D)** and the percentage of CD206^+^ BMDMs **(E)** were measured by FACS with or without eCIRP (1 µg/ml) administration for 24 h (n = 3). **(F)** A qPCR assay was conducted to evaluate the mRNA expression of EPOR and PPARγ in WT and Rab26^-/-^ BMDMs (n = 3). **(G)** The membrane EPOR of WT and Rab26^-/-^ BMDMs was evaluated by FACS (n = 3). *P < 0.05, **P < 0.01 *vs.* WT group. Data are representative of at least two independent experiments. Results were expressed as mean ± SD. n.s., not statistically significant. *P < 0.05, **P < 0.01. Statistics: One-way ANOVA with Tukey’s *post-hoc* test for multiple comparisons **(A**–**E)** or unpaired two-tailed Student’s t-test **(F, G)**. EPOR, erythropoietin receptor; BALF, bronchoalveolar lavage fluid; ALI, acute lung injury; PCR, Polymerase Chain Reaction; PPAR, peroxisome proliferator-activated receptor; BMDM, bone marrow derived macrophage; eCIRP, extracellular cold-inducible RNA-binding protein; WT, wild type; EPO, erythropoietin; MFI, mean fluorescence intensity; FACS, fluorescence activated cell sorter.

Subsequently, we explore the effect of Rab26 on macrophage phenotype *in vitro*. The level of Rab26 mRNA in BALF macrophages was suppressed at day 1, recovered at day 2, and increased by about 3-fold at days 3 and 5 (**Figure 6A**). The expression of Rab26 mRNA was suppressed by ~52.1% and ~80.2% after WT BMDMs were treated with eCIRP for 24 and 36 h, respectively (**Figure 6B**). The expression of Rab26 protein was reduced by ~30% and ~40% at 24 and 36 h, respectively (**Figure 6C**). Thus, Rab26 was involved after eCIRP treatment.

The MFIs of CD80 in Rab26 KO BMDMs were significantly increased compared with WT BMDMs (33.6 *vs.* 42.6 × 10^3^, *P* = 0.006) after rhCIRP administration for 24 h (**Figure 6D**). The baseline percentage of CD206^+^ macrophages in the WT and Rab26 KO group was similar, and the percentage was reduced after rhCIRP administration for 24 h, especially in Rab26 KO group (**Figure 6E**). Collectively, eCIRP restrained M2 macrophage polarization, especially when Rab26 was knocked out, indicating that eCIRP had the effect on macrophage polarization through Rab26.

In addition, we investigated whether Rab26 had a relationship with EPOR signaling. While the mRNA expression of EPOR did not change when Rab26 was knocked out (**Figure 6F**), the cell surface EPOR was reduced by ~35% (**Figure 6G**). The mRNA expression of PPARγ was reduced by ~45% when Rab26 was knocked out (**Figure 6F**). Collectively, Rab26 might be the critical mediator for macrophage polarization through EPOR/PPARγ signaling.

### Rab26 Deficiency Reduces Surface Erythropoietin Receptor and Restrains M2 Macrophage Polarization

**Figure 7:**
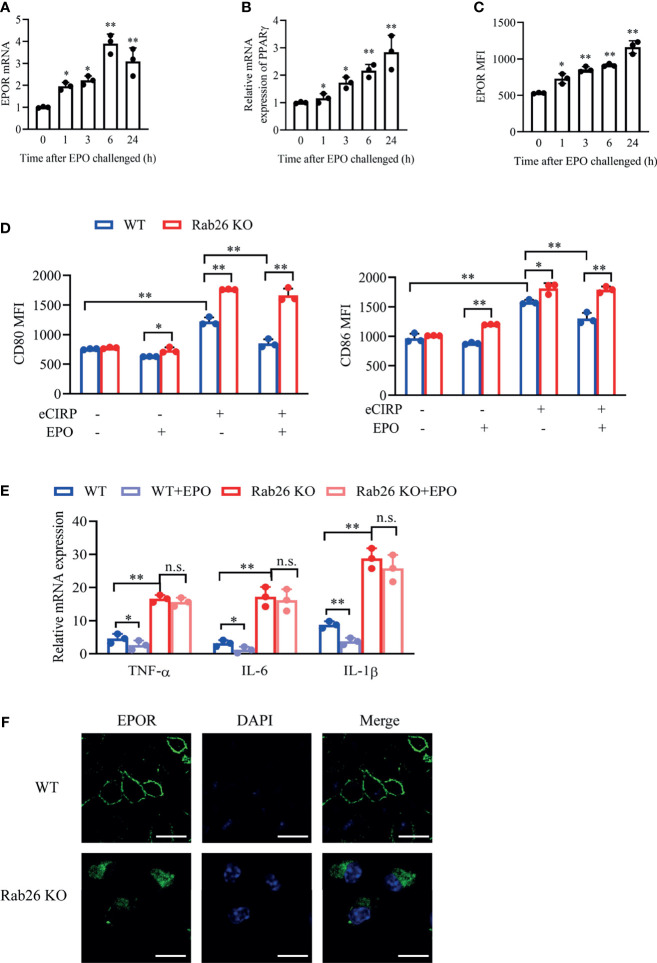
Rab26 deficiency reduces EPOR signaling and restrains macrophage polarization. A qPCR assay was conducted to evaluate the mRNA expression of EPOR **(A)** and PPARγ **(B)** in WT BMDMs treated with rhEPO (20 IU/ml) for the indicated time (n = 3). **(C)** The MFIs of cell surface EPOR in BMDMs treated with rhEPO (20 IU/ml) for the indicated times were tested by FACS (n = 3). *P < 0.05, **P < 0.01 *vs.* 0 h. **(D)** The MFIs of CD80 and CD86 were evaluated by FACS in WT and Rab26^-/-^ BMDMs treated with or without eCIRP (1 µg/ml) or rhEPO (20 IU/ml) for 24 h (n = 3). **(E)** A qPCR assay was conducted to evaluate the mRNA expression of TNF-α, IL-6, and IL-1β in WT and Rab26^-/-^ BMDMs treated with eCIRP (1 µg/ml) and rhEPO (20 IU/ml) for 24 h (n = 3). **(F)** Localization of EPOR in WT and Rab26^-/-^ BMDMs. WT and Rab26^-/-^ BMDMs were stained with an anti-EPOR antibody (1:100 dilution) and Alexa Fluor 488-labeled secondary antibody (1:200 dilution) (green), and nuclei were stained with DAPI (blue). Representative confocal images of the surface and intracellular expression of EPOR are shown. Scale bar: 10 µm. Data are representative of at least two independent experiments. Results were expressed as mean ± SD. n. s., not statistically significant. *P < 0.05, **P < 0.01. Statistics: One-way ANOVA with Tukey’s *post-hoc* test for multiple comparisons **(A–E)**. EPOR, erythropoietin receptor; PCR, polymerase chain reaction; PPAR, peroxisome proliferator-activated receptor; BMDM, bone marrow derived macrophage; EPO, erythropoietin; MFI, mean fluorescence intensity; FACS, fluorescence activated cell sorter; eCIRP, extracellular cold-inducible RNA-binding protein.

EPO could promote the mRNA expression of EPOR and PPARγ in a time-dependent way (**Figures 7A, B** **)** in WT BMDMs, and the mRNA expressions of EPOR and PPARγ were both increased by almost 3-fold at 24 h with rhEPO treatment. Similarly, the cell surface expression of EPOR was increased after EPO treatment (**Figure 7C**).

We further confirm the effect of Rab26 on EPOR location, macrophage polarization, and pro-inflammatory cytokines. The increased expression of CD80 and CD86, which was induced by rhCIRP, was reduced with rhEPO treatment in WT BMDMs, while rhEPO could not ameliorate the high expression of CD80 and CD86 in Rab26 KO BMDMs after rhCIRP administration (**Figure 7D**). The mRNA expression of TNF-α, IL-6, and IL-1β was higher in Rab26 KO BMDMs than that in WT BMDMs after rhCIRP treatment, and rhEPO ameliorated the inflammatory levels in WT BMDMs, while it worked weakly in Rab26 KO group (**Figure 7E**). Collectively, those results indicated that Rab26 deficiency restrains M2 macrophage polarization by reducing the expression of EPOR.

Furthermore, we explored the mechanism of Rab26 regulating EPOR. As revealed by confocal laser scanning microscope (CLSM), EPOR was localized mainly at the cell surface and was scarcely found in the cytoplasm in WT BMDMs, whereas it accumulated in the cytoplasm and could not be fully transported to the cell surface in Rab26 KO BMDMs (**Figure 7F**). Collectively, Rab26 regulated the cell surface expression of EPOR in BMDMs, which might be through vesicle transportation.

## Discussion

Herein, our data revealed that increased eCIRP in BALF restricted M2 macrophage polarization through EPOR/PPARγ axis, which is involved in the inflammatory response and lung tissue damage in ALI/ARDS mouse model. On the contrary, eCIRP neutralization antibody could attenuate M1 phenotype and enhance M2 phenotype in macrophages. Further studies demonstrated that eCIRP suppressed the expression of Rab26, and Rab26 deficiency reduced the surface level of EPOR through suppressing EPOR trafficking from cytoplasm to cell surface, then attenuated EPO–EPOR-mediated macrophage shifting to M2 phenotype. The novel findings show the new mechanism of eCIRP in ALI, which could be a promising therapeutic target.

eCIRP plays a critical role in mediating the inflammatory response. It is considered to activate innate Toll-like receptor (TLR)4/MD2-mediated pro-inflammatory signal (12, 25), triggering receptors expressed on myeloid cells-1 (TREM-1) and receptor for advanced glycosylation end products (RAGE) (26, 27) in hemorrhagic shock and sepsis. On the contrary, eCIRP neuralization by anti-CIRP antibody also reduced the systemic and local inflammation by attenuating serum and hepatic levels of IL-6 and reduced inducible nitric oxide synthase and cyclooxygenase 2 levels (28). Further study showed that eCIRP deficiency could reduce the number of M1 phenotype macrophage (CD68^+^/MRC1^-^) and increase the number of tissue-regenerating M2 phenotype macrophages (CD68^+^/MRC1^+^) in the ischemic tissue model (29). Consistent with documents, we also found that eCIRP shifted macrophage polarization by restraining M2 phenotype, and eCIRP neutralization antibody could partly reverse such phenotypes and attenuate the inflammatory response *in vivo*. These results indicated that eCIRP controlled the inflammatory response through regulating macrophage phenotype shifting.

An activated EPOR signaling pathway by EPO could suppress inflammatory response through promoting macrophage switch toward M2 phenotype (20, 30–32). Similar to these reports, we found that EPOR deficiency suppressed macrophage shifting to M2 phenotype and aggravated LPS-induced inflammatory response and lung tissue damage. Interestingly, our data identify a link between the eCIRP and EPOR signaling. eCIRP reduced the surface level of EPOR and suppressed EPOR downstream PPARγ, which was a regulator of macrophage polarization (33, 34). These data suggested that eCIRP might regulate macrophage polarization through EPOR/PPARγ axis.

Previous studies revealed that eCIRP could directly bind with receptors TLR4, TLR2, and RAGE and activate inflammation pathways (27). There is no piece of evidence that eCIRP could bind with EPOR directly. Therefore, we speculated that eCIRP regulated EPOR signaling indirectly, especially by regulating the level of EPOR at the cell surface.

Increasing evidence displayed some Rab GTPases, such as Rab1 and Rab26, trafficked receptors to cell surface from cytoplasm (22, 35). Our data hinted that Rab26 played a key role in trafficking EPOR to cell surface from cytoplasm, then affecting macrophage phenotype shifting. Further study displayed that eCIRP suppressed the expression of Rab26. Therefore, these data suggested that eCIRP regulated the level of EPOR at cell surface by controlling the expression of Rab26.

Based on our findings, we demonstrate the important mechanism of eCIRP on macrophage polarization and inflammatory response in ALI/ARDS. eCIRP suppressed the expression of Rab26 and then restrained M2 macrophage phenotype shifting from M1 phenotype and increased inflammatory response through suppressing EPOR/PPARγ axis. Rab26 regulated the cell surface expression of EPOR, and EPO treatment could promote macrophage M1 to M2 phenotype shifting. The results point to a potential therapeutic approach in which anti-eCIRP antibody could promote M2 macrophage polarization and reduce the inflammation in ALI/ARDS through Rab26/EPOR/PPARγ axis.

## Materials and Methods

### Reagents and Antibodies

Reagents were as follows: LPS from *Escherichia coli* O111:B4 (Sigma-Aldrich, #L4391), LPS from *Escherichia coli* 055:B5 (Sigma-Aldrich, #L2880), human CIRBP/CIRP (Sino Biological, #14578-H07E), rhEPO (Sunshine Pharmaceutical, Shenyang, China), cell dissociation buffer (Gibco, #13150016), Pierce™ BCA Protein Assay Kit (Thermo Fisher Scientific, #23225), TRIzol Reagent (Sigma-Aldrich, #T9424), cOmplete™ EDTA-free Protease Inhibitor Cocktail (Sigma-Aldrich, #04693159001), GoScript™ Reverse Transcription System (Promega, #A2800), GoTaq^®^ qPCR Master Mix (Promega, #A6001), M-PER Protein Extraction Reagent (Thermo Fisher Scientific, #78510), PageRuler Prestained Protein Ladder (Thermo Fisher Scientific, #26616), Immobilon Western Chemiluminescent HRP Substrate (Millipore, #WBKLS0500), LEGENDplex™ Multi-Analyte Flow Assay Kit (BioLegend, #740740), Immunofluorescence Application Solutions Kit (CST, #12727), Anti-fade Reagent with DAPI (Coolaber, #SL 1841), and PE Annexin V Apoptosis Detection Kit (BD, #559763).

Antibodies were as follows: APC-labeled anti-mouse F4/80 antibody (BioLegend, #123116, clone #BM8), APC/Cyanine7-labeled anti-mouse F4/80 antibody (BioLegend, #123118, clone #BM8), PE-labeled anti-mouse F4/80 antibody (BioLegend, #123110, clone #BM8), PE-labeled anti-mouse Ly-6G antibody (BioLegend, #127608, clone #1A8), APC/Cyanine7-labeled anti-mouse Ly-6G antibody (BioLegend, #127624, clone #1A8), APC/Cyanine7-labeled anti-mouse/human CD11b antibody (BioLegend, #101226, clone #M1/70), APC-labeled anti-mouse TNF-α antibody (BioLegend, #506308, clone #MP6-XT22), APC-labeled anti-mouse IL-6 antibody (BioLegend, #504508, clone #MP5-20F3), APC-labeled anti-mouse IL-10 antibody (BioLegend, #505010, clone #JES5-16E3), PE-labeled anti-mouse EPOR antibody (Bioss, #bs-1424R), FITC-labeled anti-mouse CD86 (BD, #553691, clone #GL-1), APC-labeled anti-mouse CD86 (BioLegend, #105012, clone #GL-1), PE-labeled anti-mouse CD80 (eBioscience, #12-0801-81, clone #16-10A1), PE/Cyanine7-labeled anti-mouse CD80 (BioLegend, #104734, clone #16-10A1), APC-labeled anti-mouse CD206 (BioLegend, #141707, clone #C068C2), PE-labeled anti-mouse CD206 (BioLegend, #141706, clone #C068C2), eBioscience™ Fixable Viability Dye eFluor™ 450 (Invitrogen, #65-0863-14), anti-CD16/32 antibody (Sungene Biotech, #M10161-14F); Mouse Erythropoietin R Antibody (RD, #AF1390), anti-PPARγ antibody (CTS, #2430), anti-Rab26 antibody (Abcam, #ab198202), anti-β-Actin antibody (CST, #4970S), Peroxidase AffiniPure Goat Anti-Rabbit IgG (Jackson Immunoresearch, #111-035-003), mouse anti-goat IgG-FITC (Santa Cruz, #sc-2356), and CIRP polyclonal antibody (proteintech, #10209-2-AP).

### Animal and Acute Lung Injury/Acute Respiratory Distress Syndrome Model

All animal experiments were performed in accordance with the guidelines of the Animal Care and Use Committee of the Third Military Medical University and were approved by the local Administration District Official Committee of Third Military Medical University, Chongqing, China. The generation of Rab26^-/-^ mice on a C57/BL6 background was carried out according to our previous study (22). EPOR^loxp^/^loxp^ mice on an Sv129/C57/BL6 background were backcrossed with C57/BL6 mice for more than 10 generations. Six- to eight-week-old male C57BL/6 mice were purchased from the Animal Research Center of Xinqiao Hospital affiliated with Third Military Medical University. For ALI/ARDS model induction, 3 mg/kg LPS (*Escherichia coli* 055:B5) or 1 mg/kg rhCIRP was instilled directly into the tracheas of 6- to 8-week-old mice lightly sedated with isoflurane using a modified feeding needle. Mice were sacrificed at the indicated time. Lung tissues were immediately removed, fixed with 4% paraformaldehyde, and paraffin-embedded. Paraffin-embedded tissue sections (4 μm) were observed after H&E staining for histological evaluation.

The percentage of neutrophils (Ly6G^+^F4/80^-^) in BALF at the indicated times was calculated by flow cytometry, and the total cell numbers in BALF were counted. Thus, neutrophil (Ly6G^+^F4/80^-^) numbers in BALF could be figured out during the course of ALI/ARDS, and resolution indices were calculated. The resolution of acute inflammation was defined by the following resolution indices: ψ_max_, the maximal neutrophil numbers in BALF; T_max_, the time point of maximal neutrophil numbers in BALF; R_50_, 50% of maximal neutrophil numbers; T_50_, the time point when BALF neutrophil numbers reduced to 50% of the maximum; Ri (resolution interval, T_50_–T_max_), the time period when 50% neutrophils are lost from BALF (19).

### Isolation and Cultures of Macrophages

BMDMs had been isolated according to the previous document (36). Briefly, 6- to 8-week-old C57BL/6, Rab26 KO, or EPOR-cKO mice were sacrificed, and bone marrow hematopoietic stem cells were isolated from the tibia and femur. These cells were cultured with high-glucose Dulbecco’s modified Eagle’s medium (DMEM) with 100 ng/ml recombinant macrophage colony-stimulating factor (Bioworld Technology, BK0128) and 10% fetal bovine serum for 6–7 days.

Macrophages in BALF after LPS administration were collected using anti-mouse F4/80 Micro-Beads UltraPure (Miltenyi Biotec, #130-110-443) according to the manufacturer’s instructions in order to test Rab26 mRNA.

### RNA Isolation and Real-Time PCR

Total RNA was harvested using TRIzol Reagent, and 1 μg total RNA was reverse transcribed into cDNA using GoScript™ Reverse Transcription System. The cDNA was used to measure the relative expression of genes using GoTaq^®^ qPCR Master Mix. Gene expression was presented as the glyceraldehyde-3-phosphate dehydrogenase (GAPDH) normalized. The primers are listed as follows: Rab26 forward, 5′-ACTCTACTCAAGACCGTGTGG-3′; reverse, 5′-TCCATGAAAGGTAGCCCATACT-3′; EPOR forward 5′-GGTGAGTCACGAAAGTCATGT-3′, reverse 5′-CGGCACAAAACTCGATGTGTC-3′; PPARγ forward 5′-TCGCTGATGCACTGCCTATG-3′, reverse 5′-GAGAGGTCCACAGAGCTGATT-3′; CD206 forward 5′-CTCTGTTCAGCTATTGGACGC-3′, reverse 5′-TGGCACTCCCAAACATAATTTGA-3′; CD86 forward 5′-GAGCTGGTAGTATTTTGGCAGG-3′, reverse 5′-GGCAGGTACTTGGCATT-3′; Arg1 forward 5′-CATATCTGCCAAAGACATCGTG-3′, reverse 5′-GACATCAAAGCTCAGGTGAATC-3′; CD80 forward 5′-CAACTGTCCAAGTCAGTGAAAG-3′, reverse 5′-CACCACTTTGTCATGTTTTTGC-3′; TNF-α forward, 5′-GACCCTCACACTCAGATCATC-3′, reverse, 5′-GAACCTGGGAGTAGATAAGG-3′; IL-6 forward, 5′-GTATGAACAACGATGATGCACTTG-3′, reverse, 5′-ATGGTACTCCAGAAGACCAGAGGA-3′; IL-10 forward, 5′-GCTCTTACTGACTGGCATGAG-3′, reverse, 5′-CGCAGCTCTAGGAGCATGTG-3′; IL-1β forward, 5′-GCTCTTACTGACTGGCATGAG-3′, reverse, 5′-CGCAGCTCTAGGAGCATGTG-3′; CIRP forward, 5′-AGGGTTCTCCAGAGGAGGAG-3′, reverse, 5′-CCGGCTGGCATAGTAGTCTC-3′; GAPDH forward, 5′-GGGAGCCAAAAGGGTCAT-3′, reverse, 5′-GAGTCCTTCCACGATACCAA-3′.

### Collection of Bronchoalveolar Fluid

The lungs were lavaged three times with 500 μl of sterile cold PBS (total volume 1.5 ml) to collect BALF at the indicated time after LPS, rhCIRP, or PBS administration. A total volume of 1.3 ml was recovered. The BALF samples were centrifuged at 300 g for 10 min at 4°C, and the cell-free supernatants were maintained at -80°C to determine the cytokine and protein concentrations. The cells from BALF were kept at 4°C for further flow cytometry.

### ELISA

BALF was used to detect the concentrations of eCIRP (MEIMIAN, #45627M1) using an ELISA kit according to the manufacturer’s instructions.

### Flow Cytometry

The cells were blocked with anti-CD16/32 for 20 min to reduce non-specific binding. For the analysis of EPOR, CD80, CD86, and CD206 levels at the cell surface, harvested cells were incubated with anti-EPOR, anti-CD86, anti-CD80, anti-CD206 antibody, and anti-F4/80 antibody on ice for 30 min, washed twice, and then taken to flow cytometry. For the analysis of intracellular TNF-α, IL-6, and IL-10 levels, harvested cells in BALF from eCIRP-treated mice were incubated with anti-F4/80 antibody for 30 min. Subsequently, the cells were permeabilized and fixed with Cytofix/Cytoperm™ Fixation/Permeabilization kit (BD, #554714) and further stained with anti-TNF-α, anti-IL-6, and anti-IL-10 antibody on ice for 30 min, washed twice, and then taken to flow cytometry. Flow cytometry was performed using a FACScan cytometer CANTO II (Becton Dickinson, Franklin Lakes, NJ, USA) or Gallios (Beckman Coulter, USA). Data were collected using CellQuest software (Becton Dickinson) or Kaluza software (Beckman coulter) and analyzed with FlowJo software (Tree Star, Inc.). Macrophages in BALF were marked by F4/80, and the MFIs and percentage of target proteins were measured.

### Immunofluorescence Staining

BMDMs were grown in glass bottom cell culture dishes, fixed with 4% paraformaldehyde, and then conducted under the instructions of the Immunofluorescence Application Solutions Kit (CST, #12727). Cells in the dishes were stained with primary antibodies and appropriate fluorescence-conjugated secondary antibodies. The cells were viewed with a Leica confocal microscope (Leica Microsystems, Germany) and analyzed using ImageJ software (NIH, USA) or Leica LAS AF 2.3.0 software (Leica Microsystems, Germany).

### The Detection of the Level of Inflammatory Cytokines

The culture supernatants of treated BMDMs and BALF were collected at the indicated times after LPS or rhCIRP administration. These samples were analyzed using LEGENDplex™ Multi-Analyte Flow Assay Kit to determine concentrations of inflammatory cytokines, including TNF-α, IL-6, and IL-10, according to the manufacturer’s instruction (17).

### Western Blot

Samples were extracted from cells using M-PER reagent. The protein concentrations were detected through bicinchoninic acid (BCA) assay kit (Thermo Fisher Scientific, #23225). Western blotting was performed as reported (37). In brief, protein samples were electrophoresed in 8%–15% sodium dodecyl sulfate polyacrylamide gel electrophoresis (SDS-PAGE), incubated with the appropriate antibodies and measured by ChemiDoc™ MP imaging system (Bio-Rad, USA). The densitometric analysis was conducted using ImageJ software.

### Survival Rate Assay

C57BL/6, Rab26 KO, and EPOR-cKO mice were challenged with 10 mg/kg LPS intra trachea (i.t.), and then the survival percentage in each group was monitored for 7 days.

### Lung Histology and Lung Injury Scoring

Mice were sacrificed at the indicated time after LPS or eCIRP administration, and the lungs were fixed by instillation of 4% paraformaldehyde and embedded in paraffin. Lung tissue sections were stained with H&E.

The severity of lung injury was evaluated by three blinded investigators according to the histological semiquantitative scoring system (38). Each investigator scored all lung fields per slide at ×20 magnification. Points were given for each field as the following criteria: 1, normal; 2, focal (<50% lung section) interstitial congestion and inflammatory cell infiltration; 3, diffuse (>50% lung section) interstitial congestion and inflammatory cell infiltration; 4, focal (<50% lung section) consolidation and inflammatory cell infiltration; and 5, diffuse (>50% lung section) consolidation and inflammatory cell infiltration. The mean score was used for comparison between groups.

### Statistical Analysis

Quantification of replicate experiments was presented as the mean ± SD, and the experiments were conducted at least three times. The difference between the two groups was statistically analyzed by unpaired two-tailed Student’s t-test when the data meet the normal distribution criteria and analyzed by nonparametric test when the data did not assume a Gaussian distribution. The differences among three or more groups were statistically analyzed by analysis of variance (one-way ANOVA), and log-rank tests were applied to determine statistical significance for survival rate, with *P* values set to less than 0.05.

## Data Availability Statement

The raw data supporting the conclusions of this article will be made available by the authors without undue reservation.

## Ethics Statement

The animal study was reviewed and approved by the Laboratory Animal Welfare and Ethics Committee of Third Military Medical University.

## Author Contributions

WZ, YW, CL, and BH contributed to the experimental design, performed research, analyzed data, and wrote the first draft of the article. BH and HQ wrote and revised the article. YX, XW, DW, ZG, and ZY performed some in vitro experiments. GW, ZZ, and BH contributed to the study concept, research design, data analysis, and finalized the article. All authors contributed to the article and approved the submitted version.

## Funding

This work was supported by the National Natural Science Foundation of China (Nos. 81800086, 81670047, 81873413, and 82070071) and the Natural Science Foundation of Chongqing (Nos. cstc2021ycjh-bgzxm0011, cstc2019jcyj-msxmS0367).

## Conflict of Interest

The authors declare that the research was conducted in the absence of any commercial or financial relationships that could be construed as a potential conflict of interest.

## Publisher’s Note

All claims expressed in this article are solely those of the authors and do not necessarily represent those of their affiliated organizations, or those of the publisher, the editors and the reviewers. Any product that may be evaluated in this article, or claim that may be made by its manufacturer, is not guaranteed or endorsed by the publisher.

## Supplementary Material

Supplementary Figure 1 eCIRP is temporally activated in acute lung injury. **(A)** The gating strategy for BALF macrophages to analyze the MFIs of CD80 and define CD206^+^ M2 macrophage populations *in vivo*. **(B)** A qPCR assay was conducted to evaluate the mRNA expression of CD80, CD86, Arg1 and CD206 in BMDMs after eCIRP treatment (0, 0.1, 1, 10 µg/mL) for 24 h (n=3). **(C)** A qPCR assay was conducted to evaluate the mRNA expression of TNF-α, IL-6, and IL-1β in BMDMs after eCIRP treatment (0, 0.1, 1, 10 µg/mL) for 24 h (n=3). Data are representative of at least two independent experiments. Results were expressed as mean ± SD. **P* < 0.05, ***P* < 0.01 *versus* the WT group. Statistics: One-way ANOVA with Tukey’s *post hoc* test for multiple comparisons **(B, C)**.

Supplementary Figure 2 Myeloid EPOR deficiency aggravates inflammation in lung injury. **(A)** WT and EPOR cKO mice (n=6) were treated with 3 mg/kg LPS (i.t.) at the indicated times (days 0, 1, 2, 3 and 5). The time course of neutrophils (Ly6G^+^F4/80^-^) numbers in BALF and resolution indices was calculated by flow cytometry. **(B)** The number of annexin V^+^ neutrophils in BALF was calculated after LPS administration (n=4). Data are representative of at least two independent experiments. Results were expressed as mean ± SD. **P* < 0.05, ***P* < 0.01 *versus* the WT group. Statistics: unpaired two-tailed Student’s t-test **(B)**.
