# Supplementary figures and images for: Extracellular CIRP-Impaired Rab26 Restrains EPOR-Mediated Macrophage Polarization in Acute Lung Injury
Source: Front Immunol. 2021 Dec 1;12:768435. doi: 10.3389/fimmu.2021.768435 (PMC8671298; doi:10.3389/fimmu.2021.768435)

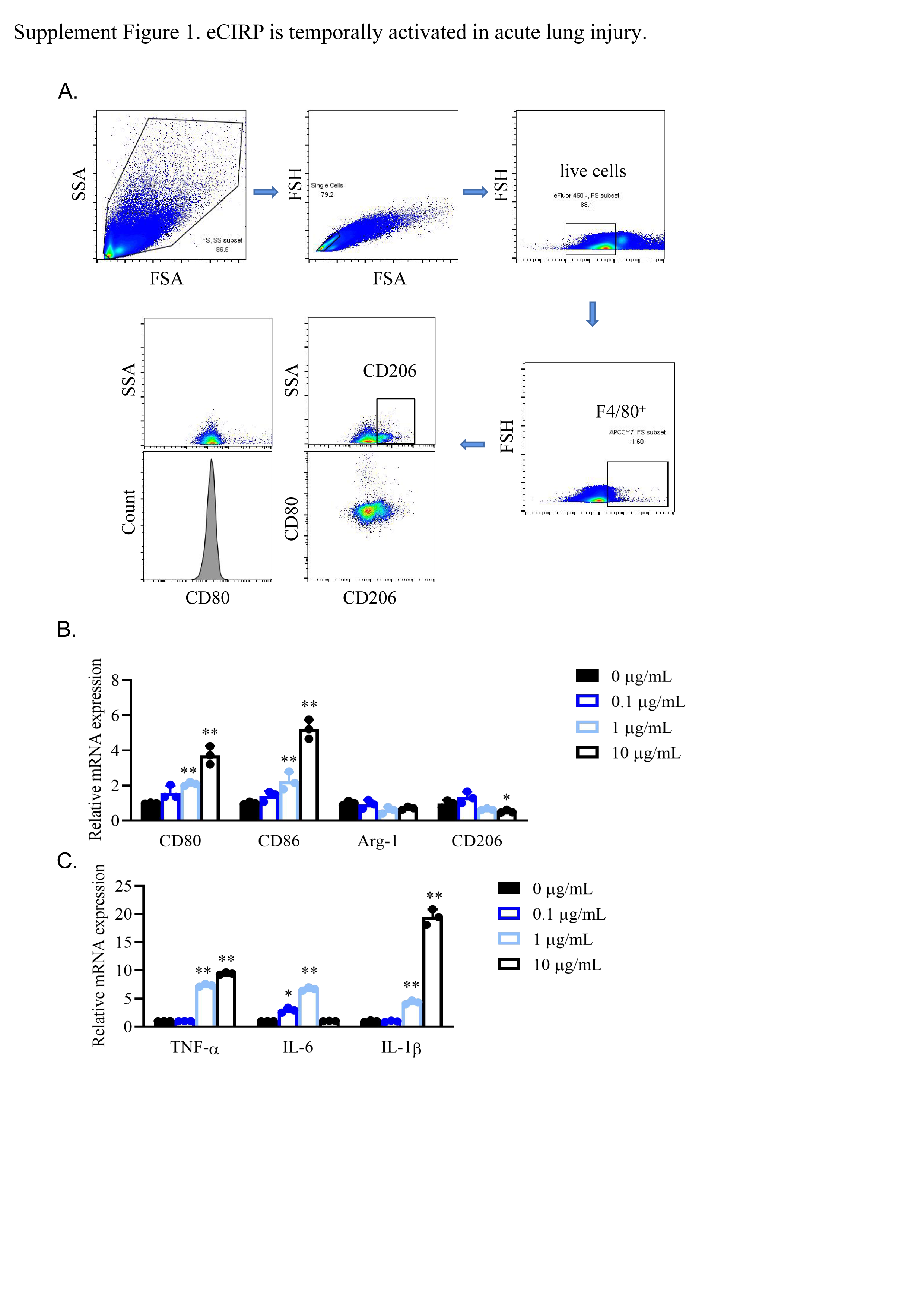

Supplement: Supplementary Figure 1 — eCIRP is temporally activated in acute lung injury. (A) The gating strategy for BALF macrophages to analyze the MFIs of CD80 and define CD206+ M2 macrophage populations in vivo. (B) A qPCR assay was conducted to evaluate the mRNA expression of CD80, CD86, Arg1 and CD206 in BMDMs after eCIRP treatment (0, 0.1, 1, 10 µg/mL) for 24 h (n=3). (C) A qPCR assay was conducted to evaluate the mRNA expression of TNF-α, IL-6, and IL-1β in BMDMs after eCIRP treatment (0, 0.1, 1, 10 µg/mL) for 24 h (n=3). Data are representative of at least two independent experiments. Results were expressed as mean ± SD. *P < 0.05, **P < 0.01 versus the WT group. Statistics: One-way ANOVA with Tukey’s post hoc test for multiple comparisons (B, C). [file Image_1.tif]

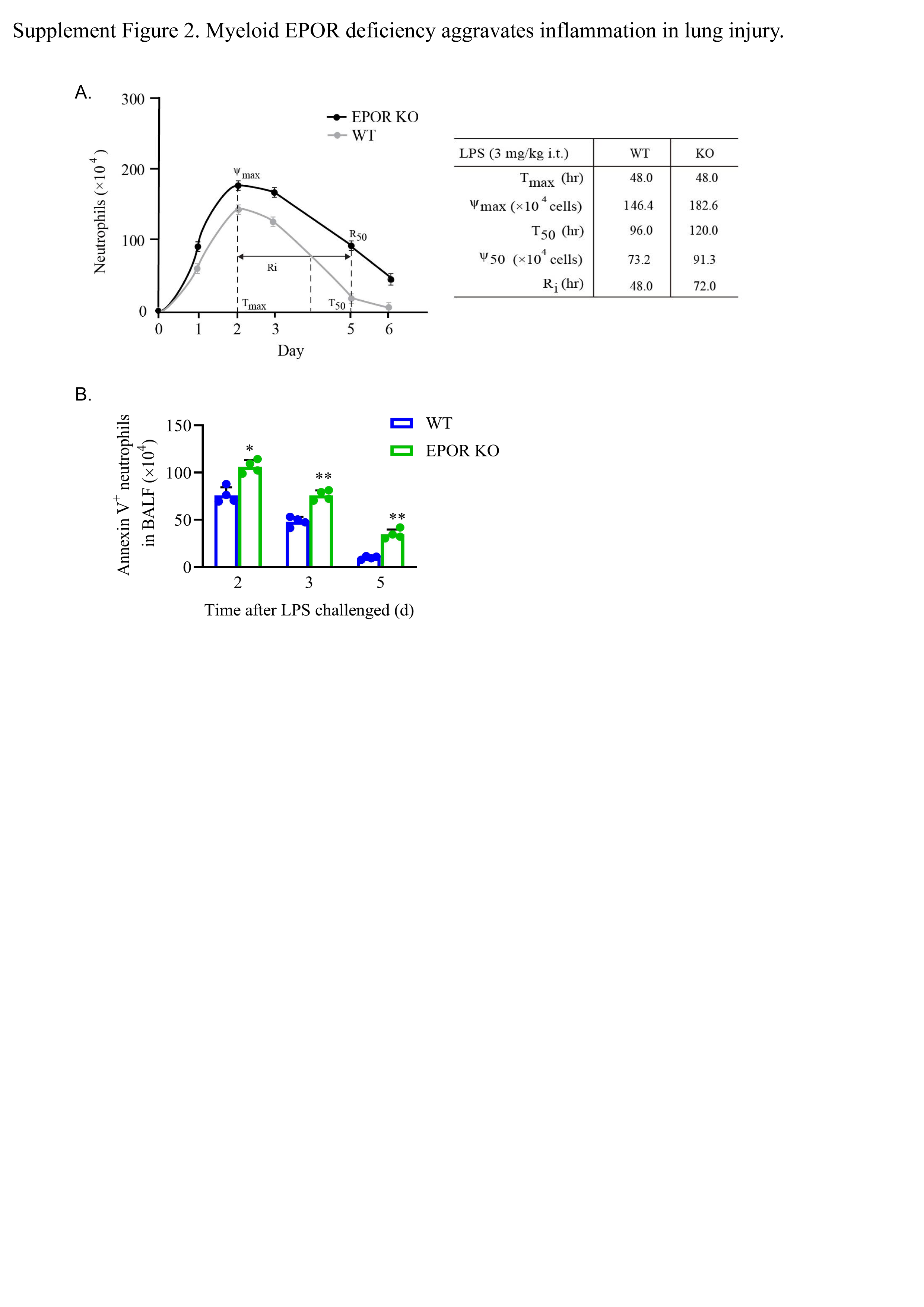

Supplement: Supplementary Figure 2 — Myeloid EPOR deficiency aggravates inflammation in lung injury. (A) WT and EPOR cKO mice (n=6) were treated with 3 mg/kg LPS (i.t.) at the indicated times (days 0, 1, 2, 3 and 5). The time course of neutrophils (Ly6G+F4/80-) numbers in BALF and resolution indices was calculated by flow cytometry. (B) The number of annexin V+ neutrophils in BALF was calculated after LPS administration (n=4). Data are representative of at least two independent experiments. Results were expressed as mean ± SD. *P < 0.05, **P < 0.01 versus the WT group. Statistics: unpaired two-tailed Student’s t-test (B). [file Image_2.tif]
